# Case Report: Cervical carbon fiber-reinforced PEEK pedicle screw fixation with 3D custom-made template guides for spinal oncology surgery

**DOI:** 10.3389/fsurg.2025.1725931

**Published:** 2026-01-28

**Authors:** Fabio Cofano, Nicola Marengo, Stefano Colonna, Francesca Rizzo, Filippo Lacatena, Diego Garbossa

**Affiliations:** Neurosurgery Unit, Department of Neuroscience “Rita Levi-Montalcini”, University of Turin, Turin, Italy

**Keywords:** cervical CFR-PEEK pedicle screw, CFR-PEEK, 3D template guides, spinal surgery, spinal oncology, stereotactic radiosurgery

## Abstract

Cervical spine fixation in oncological and tumor-like conditions poses unique mechanical and radiological challenges. The choice of implant material is crucial to ensure optimal biomechanical stability and minimize imaging artifacts that may hinder adjuvant radiotherapy. While traditional titanium constructs provide reliable mechanical performance, they generate significant postoperative artifacts. Carbon fiber–reinforced PEEK (CFR-PEEK) implants offer comparable biomechanical strength with markedly reduced imaging interference, facilitating radiotherapy planning. However, their application in the cervical spine remains extremely limited due to narrow anatomical corridors. This technical note reports the first use of CFR-PEEK cervical pedicle screws (CPS) guided by patient-specific 3D-printed templates in the treatment of an aggressive vertebral hemangioma. The study demonstrates the technical feasibility and clinical applicability of CFR-PEEK screws with customized 3D guides for posterior cervical fixation in complex oncologic cases.

## Introduction

Spinal fixation in oncological and tumor-like conditions presents specific mechanical and radiological challenges, particularly in the cervical region, where anatomical complexity and biomechanical needs are high. The choice of implant material is crucial in patients who may require frequent and extensive postoperative imaging and adjuvant radiotherapy. Traditional titanium constructs, although mechanically reliable, produce significant artifacts on CT and MRI and can interfere with radiotherapy planning or obscure local recurrence assessment (1–3).

Carbon fiber–reinforced polyetheretherketone (CFR-PEEK) implants have recently emerged as a valid alternative to metallic systems. These composite materials provide adequate mechanical strength, an elastic modulus closer to cortical bone, and excellent fatigue resistance, while their radiolucency allows clear visualization of bone–implant interfaces and tumor margins (4, 5). Their favorable imaging properties also allow more accurate radiotherapy planning and dose calculation and postoperative surveillance (6). Clinical studies have confirmed their safety and stability, particularly in thoracic and lumbar reconstructions, where comparable outcomes to titanium systems have been found with superior imaging and radiotherapy compatibility (7–10).

Despite these advantages, the application of carbon fiber–based fixation in the cervical spine remains extremely limited due to smaller anatomical corridors and the need for high mechanical precision. To date, no previous report has described the use of carbon fiber screws for fixation in the cervical region. In this technical note, we present the first known case of cervical spine fixation using carbon fiber–reinforced screws for the treatment of an aggressive vertebral hemangioma. We describe the surgical strategy, technical nuances of screw placement, and postoperative outcomes, highlighting the feasibility and potential benefits of this material in cervical spine.

## Case description

A 74-year-old woman presented with a progressively worsening paraparesis that had developed insidiously over the previous four years. Given the gradual but significant decline in lower limb strength and function, she was referred for a neurosurgical evaluation. Clinical assessment raised the suspicion of a compressive spinal lesion, prompting the indication for a complete spinal MRI. The MRI revealed an area of altered signal within the D3 vertebral body, with radiological features suggestive of a vertebral hemangioma. Notably, the lesion demonstrated infiltration of the posterior epidural space and anterior bilateral paravertebral region, raising concern for extraosseous extension and aggressive behavior. To better define the lesion's characteristics and extent, a contrast-enhanced MRI of the cervico-thoracic spine was performed. This confirmed the presence of a morpho-structural alteration involving both the vertebral body and posterior elements of D3. Additionally, pathological epidural and paravertebral tissue extended from D1 to D4 on the right side, causing evident compression of the spinal cord and radiological signs of myelopathy. The imaging findings were compatible with an aggressive vertebral hemangioma with extraosseous growth. To further characterize the lesion, a cervico-thoracic CT scan was obtained, which supported the suspicion of a hypervascular, structurally disruptive lesion centered at D3.

**Figure 1:**
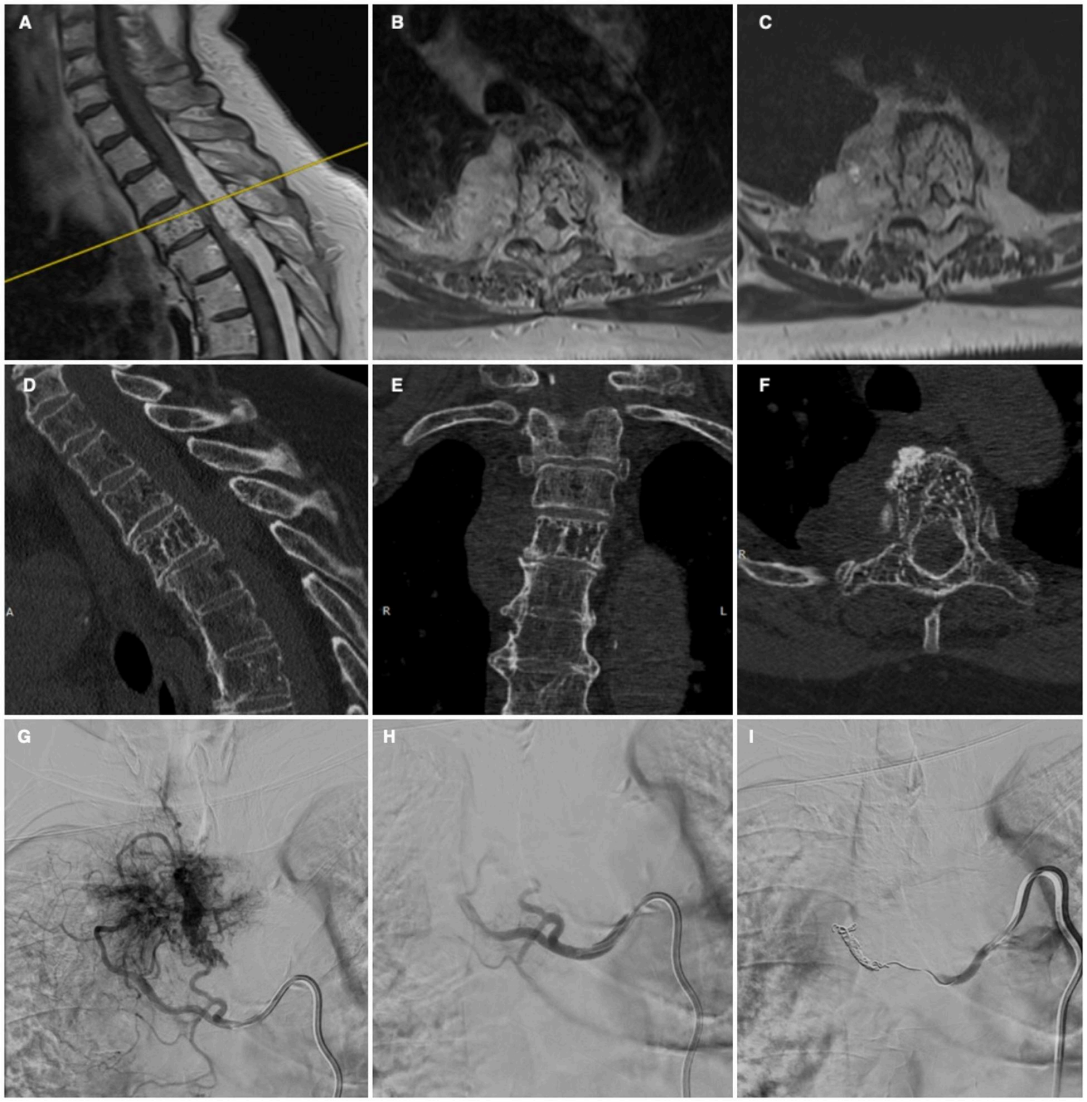
Sagittal **(A)** and axial **(B,C)** cervical-thoracic MRI images showing morpho-structural alteration of the D3 vertebral body with posterolateral involvement, associated with epidural and paravertebral pathological tissue causing compressive myelopathy. Cervical-thoracic CT images **(D–F)** confirming a hypervascular lesion of the D3 vertebral body with osteostructural disruption, consistent with hemangioma. Spinal angiography confirming hypervascular D3 lesion with arterial supply originating from the right D3-D4 metameric artery **(G)**, and subsequent selective embolization using polyvinyl alcohol microparticles and microcoils **(H,I)**.

Considering these findings, a multidisciplinary discussion was held in September 2025 involving neurosurgery and interventional neuroradiology teams. Given the extent of the lesion, spinal cord compression, and the patient's progressive neurological symptoms, a combined treatment approach was recommended. Subsequently, the patient underwent spinal angiography, which confirmed a lesional arterial supply originating from the right D3-D4 metameric artery. A preoperative embolization was performed with selective catheterization of the feeder vessel, followed by injection of polyvinyl alcohol (PVA) microparticles and placement of controlled release microcoils. Embolization achieved a satisfactory occlusion of the feeding vessels, forming a dense vascular cast and significantly reducing the lesion's vascularity (Figure 1).

The following day, the patient underwent surgical intervention. A posterior cervico-thoracic fixation was performed using patient-specific, 3D-printed surgical guides (Medacta MySpine system), allowing for precise placement of carbon fiber pedicle screws at C6, C7, unilateral D4, D5, and D6.

**Figure 2:**
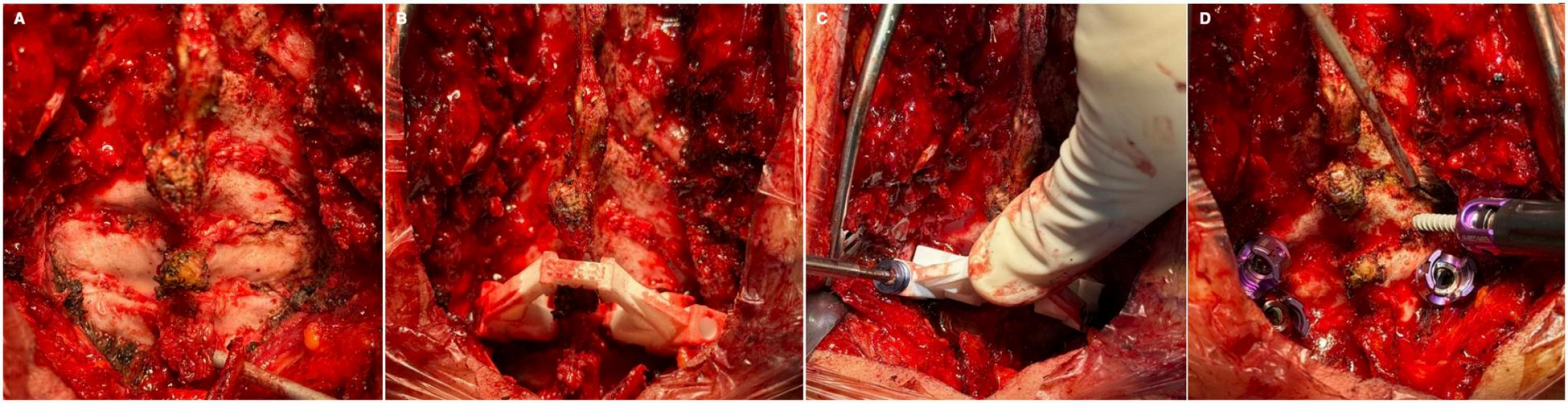
Intraoperative images demonstrating accurate removal of soft tissue from bony landmarks, maximizing the adherence of the template to the dorsal surface of the vertebrae **(A,B)**. Once the template is firmly secured to the vertebrae, a drill bit is used to delineate the pedicle trajectory according to the preoperative surgical plan before placing the screw **(C,D)**.

Preoperative planning was based on a high-resolution cervical CT scan with sub-millimetric slice thickness (<1 mm). Two senior spine surgeons independently reviewed the imaging to exclude anatomical configurations incompatible with safe pedicle screw placement, including pedicle transverse angles >45°, pedicle width <3.5 mm, insufficient cancellous channel, and vertebral artery variants involving the pedicle or vertebral body. Patient-specific guides were then generated from the CT dataset. A dedicated planning platform (MySpine Surgical Planning Report, Medacta, Rancate, Switzerland) was used to define the optimal screw entry point, trajectory, and dimensions. Guides were designed to achieve secure, anatomy-matched contact with the dorsal vertebral surface through two cylindrical support interfaces, ensuring intraoperative stability (Figure 2). Templates were manufactured in Polyamide-PA12 using 3D printing and sterilized preoperatively according to the validated protocol. This well-known technology was matched with the introduction in the global market of 4.5 diameter cannulated carbon fiber-reinforced PEEK pedicle screws (Icotec, Altstätten, Switzerland), that enabled cervical pedicle screwing.

**Figure 3:**
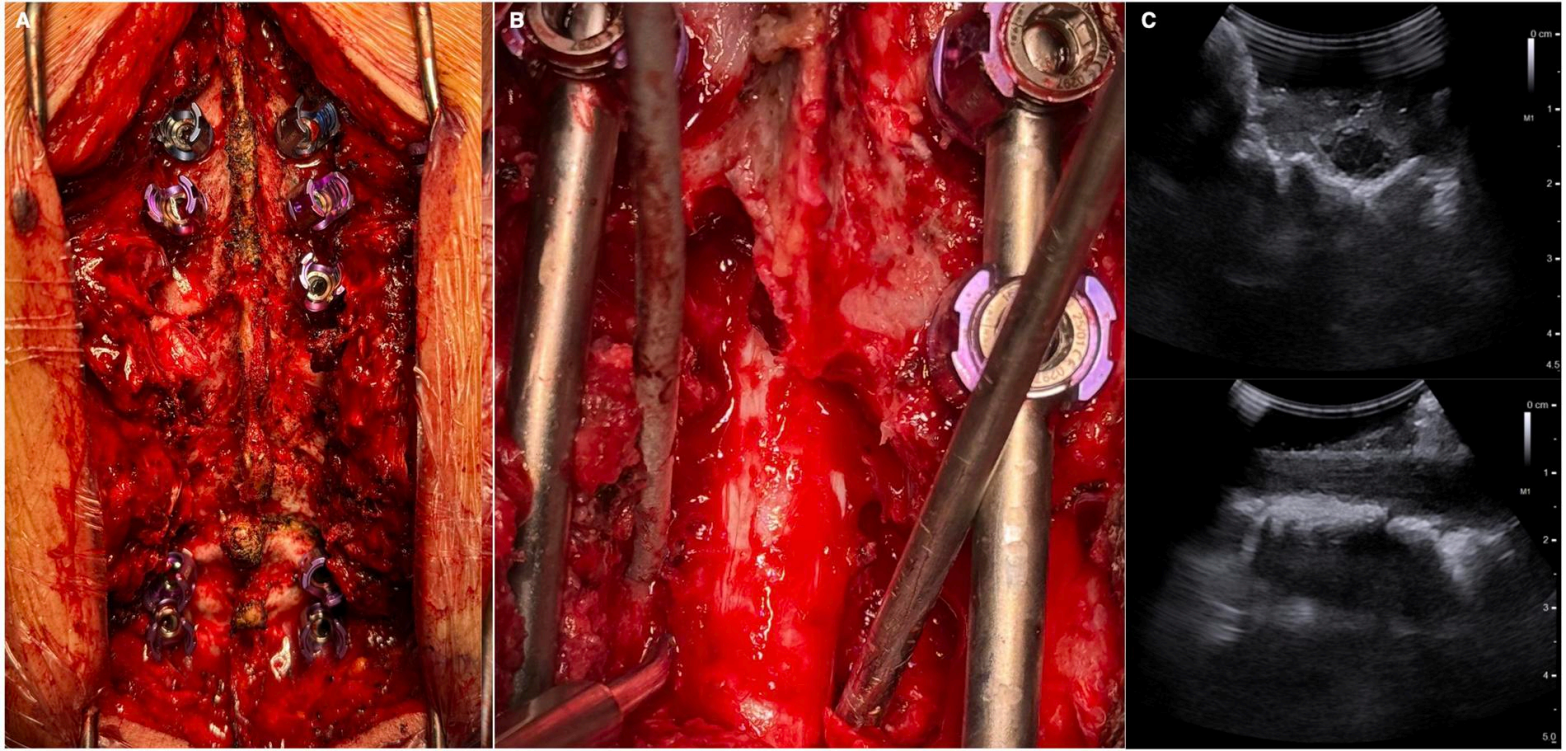
Intraoperative images demonstrating posterior C6-D6 fixation using CFR-PEEK pedicle screws **(A)** close-up image of D3 posterior right-transpedicular circumferential decompression **(B)**, with intraoperative ultrasound confirming satisfactory relief of cord compression **(C)**.

A transpedicular approach for circumferential decompression and separation surgery was carried out at D3, consisting of bilateral facetectomy and pediculectomy, followed by vertebroplasty of the affected vertebral body using bone cement. This approach allowed for decompression of the spinal canal and partial debulking. Intraoperatively, angiomatoid tissue compressing the thecal sac was meticulously removed, including the ventral components encasing the spinal cord. The extent of decompression was verified with intraoperative ultrasound, which confirmed satisfactory relief of cord compression and reconstitution of the thecal sac. Posterior decompression was enlarged at the levels above and below according to the extent of the disease (Figure 3).

**Figure 4:**
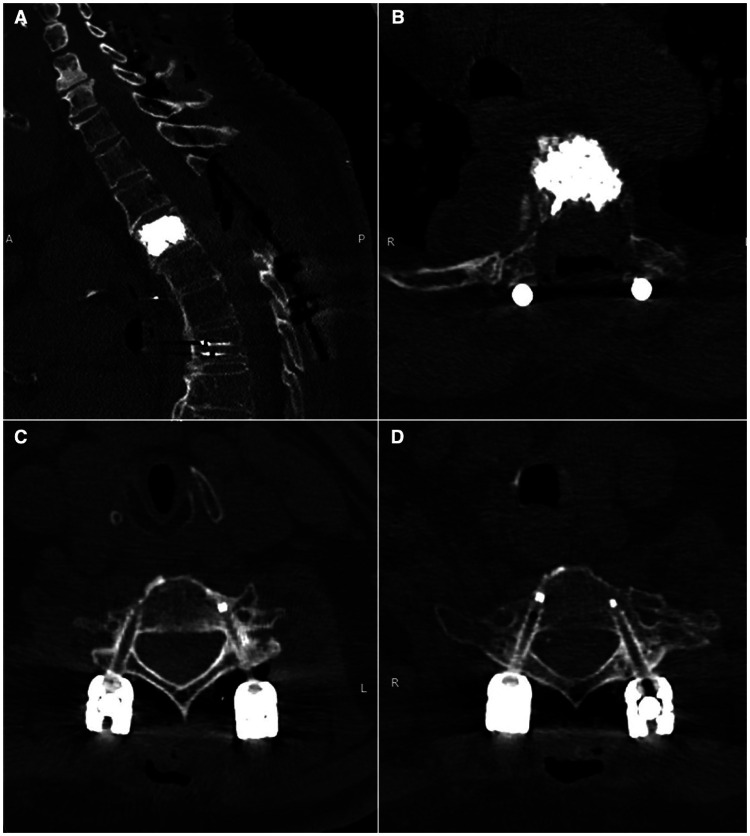
Postoperative CT scan demonstrating effective decompression with D3 vertebroplasty **(A,B)**. Axial CT images confirming Gertzbein grade A C6-C7 pedicle screws **(C,D)**.

The postoperative course remained uneventful. The patient began cautious mobilization with early motor improvement, and a cervical-thoracic CT scan at one month confirmed optimal positioning of all screws (Gertzbein grade A) and satisfactory vertebroplasty at D3 (Figure 4). Neurological examination showed no new deficits compared with the preoperative assessment, with initial improvement of the preexisting gait disturbance. Following multidisciplinary consultation with radiation oncologists, stereotactic radiosurgery (SRS) was deferred and considered as a potential option should radiological evidence of tumor progression emerge during subsequent follow-up. Clinical and radiological follow-up is ongoing, with progressive improvement in gait function.

## Discussion

The management of spinal tumors has evolved substantially over the past decades, driven by advances in oncologic therapies and continuous improvements in surgical technology. In parallel, major progress in radiotherapy has transformed the natural history and post-treatment outcomes of these diseases (11–15). Today, adjuvant radiotherapy is a cornerstone of spinal tumor management (16–18). However, the accuracy of radiotherapy depends not only on modern treatment platforms but also on the quality of postoperative imaging used for planning (19–22).

The introduction of CFR-PEEK implants has brought meaningful benefits to spinal tumor surgery. Both *in vitro* and clinical studies have demonstrated that CFR-PEEK instrumentation is biomechanically comparable to titanium systems in the thoracolumbar spine, with similar bending yield load, axial compression strength, and bending stiffness (6, 23–27). In a systematic review by Khan et al., the implant-related complication rate with CFR-PEEK was 7.8%, comparable to titanium constructs (28). Similarly, in a recent case series of 190 patients treated with CFR-PEEK instrumentation, mechanical implant failure occurred in 4.1% of cases at a median follow-up of 13.7 months, consistent with previously reported titanium outcomes (29).

Beyond mechanical performance, CFR-PEEK implants offer major advantages for adjuvant radiotherapy. The low radiological artifact profile allows for more accurate target definition and better optimization of treatment volumes in both photon and proton therapy (1, 2, 8). Reduced imaging distortion also shortens radiotherapy planning correction time and significantly minimizes discrepancies between planned and delivered doses (7, 30).

While CFR-PEEK constructs are well established in thoracolumbar oncologic surgery, their application in the cervical spine remains scarcely documented. Previous reports – most notably by Boriani et al. – described hybrid configurations in which thoracic CFR-PEEK rods were coupled with titanium cervical sublaminar bands positioned outside the irradiation field, achieving favorable outcomes but without addressing the feasibility of a fully carbon-based cervical screw construct (31, 32). To date, no study has reported posterior cervical fixation performed entirely with CFR-PEEK screws, nor the clinical use of CPS manufactured solely from this material.

The present case extends the clinical applicability of CFR-PEEK technology by demonstrating the feasibility of a fully CFR-PEEK posterior cervical screw construct, made possible by the recent development of small-diameter CPS specifically engineered for the cervical spine. This represents a meaningful advance compared with prior hybrid approaches, particularly in the context of spinal oncology where radiotrasparency and artifact reduction are critical.

Compared to lateral mass screws, CPS provide significant biomechanical advantages in terms of pull-out strength and primary stability. After uniplanar cyclic loading, pull-out strengths of 1,214 N vs. 332 N have been reported for CPS and LMS, respectively. Moreover, CPS reached 762 N of pull-out strength compared to 191 N for LMS under torsional loading, and 571 N vs. 289 N in flexion-extension (33, 34). These advantages are particularly valuable in spinal oncology, where bone quality often is compromised and long-term construct stability is essential.

CPS placement remains technically demanding, but recent advances have significantly improved accuracy and safety (35–37). In the present case, CPS were inserted using patient-specific 3D-printed guides designed from a high-resolution volumetric preoperative CT scan. Several studies have demonstrated the safety and reliability of this technique, with accuracy rates ranging from 93% to 100% (38–44). The main advantage of using 3D-printed guides lies in their collinearity with the dorsal surface of the vertebrae. Accurate soft tissue dissection and patient-specific customized templates ensure optimal stability of the guide on the vertebral surface, significantly minimizing inaccuracies related to vertebral rotation during pedicle cannulation and screw placement as described for the use of neuronavigation. Moreover, the use of unilateral guides markedly reduces paravertebral muscle retraction, lowering the risk of a muscle-pushing effect that could lead to suboptimal lateral trajectories with potential vertebral artery injury.

CPS can also be placed freehand or with intraoperative navigation assistance; however, a recent multicenter comparative study demonstrated that 3D guide–assisted placement achieved significantly higher accuracy and lower complication rates than navigation-assisted placement (97.7% vs. 85.5%, *p* < 0.001) (45). Moreover, these findings were confirmed by a recent meta-analysis by Bindels et al., which demonstrated no improvement in screw placement accuracy when using navigation-assisted techniques (46). The lower accuracy may be attributed both to vertebral rotation during pedicle cannulation, which is not detected by the navigation system, and to even minimal incidental shifts of the reference arrays during surgical maneuvers. In support of this hypothesis, accuracy using navigation-assisted techniques tends to decrease in the mid-cervical segments (C3–C5), where vertebral mobility and rotational potential are greater compared to the more caudal levels (C6–C7) (47–49).

### Limitations

The main limitation of this technique is the limited availability of screw sizes, because there is still lack of screws with less than 4.5-mm diameter produced. This means that this technique is not applicable in cases with thin cervical pedicles. Furthermore, given the time required for guide fabrication and delivery (5–10 days), the approach is also unsuitable for urgent cases due to the time required for guide fabrication. Finally, CFR-PEEK implants are substantially more expensive than titanium systems, making careful patient selection essential to maximize the cost–benefit ratio.

## Conclusions

This study demonstrates the technical feasibility and clinical applicability of CFR-PEEK cervical pedicle screws for posterior fixation in oncologic surgery, supported by the precision of 3D-printed patient-specific guides. Although current limitations in screw sizing, production time, and cost restrict its widespread adoption, this approach may pave the way for broader use of radiolucent implants in complex cervical and oncologic spine surgery.

## Data Availability

The raw data supporting the conclusions of this article will be made available by the authors, without undue reservation.
